# Molecular characterization of emerging variants of PRRSV in the United States: new features of the -2/-1 programmed ribosomal frameshifting signal in the nsp2 region

**DOI:** 10.1016/j.virol.2022.06.004

**Published:** 2022-06-05

**Authors:** Xingyu Yan, Pengcheng Shang, Wannarat Yim-im, Yankuo Sun, Jianqiang Zhang, Andrew E. Firth, James Lowe, Ying Fang

**Affiliations:** 1 Department of Pathobiology, College of Veterinary Medicine, University of Illinois, Urbana-Champaign, Urbana, IL, USA; 2 Department of Diagnostic Medicine and Pathobiology, College of Veterinary Medicine, Kansas State University, Manhattan, KS, USA; 3 Veterinary Diagnostic and Production Animal Medicine, Iowa State University, Ames, Iowa, USA; 4 Department of Pathology, University of Cambridge, Cambridge, U.K.; 5 Department of Veterinary Clinical Medicine, College of Veterinary Medicine, University of Illinois, Urbana-Champaign, Urbana, IL, USA

## Abstract

In this study, we characterized an emerging porcine reproductive and respiratory syndrome virus (PRRSV) isolate UIL21-0712, which is a lineage 1C variant with ORF5 restriction fragment length polymorphism (RFLP) cutting pattern of 1-4-4. The UIL21-0712 genome sequence has 85.3% nucleotide identity with the prototypic PRRSV-2 strain VR2332. The nsp2 region is the most variable, and the -2/-1 programmed ribosome frameshifting (PRF) signal therein is distinct from historical PRRSV strains. Analysis of PRRSV sequences in GenBank revealed that the majority of the emerging PRRSV variants contain substitutions that disrupt the -1 PRF stop codon to generate a nsp2N protein with a C-terminal extension. Two of the -1 PRF stop codon variant patterns were identified to be predominantly circulating in the field. They demonstrated higher growth kinetics than the other variants, suggesting that the most dominant -1 PRF stop codon variant patterns may provide enhanced growth fitness for the virus.

## 1 Introduction

Porcine reproductive and respiratory syndrome virus (PRRSV) is the etiological agent of porcine reproductive and respiratory syndrome (PRRS), characterized by respiratory diseases in growing pigs and reproductive failure in sow (Rossow, 1998). PRRSV is an enveloped, positive-stranded RNA virus, which belongs to the order *Nidovirales*, family *Arteriviridae* (Snijder et al., 2013). Historically, PRRSV isolates have been divided into two distinct genotypes, European genotype (Type 1, PRRSV-1) and North American genotype (Type 2, PRRSV-2), which were recently reclassified into two species, designated as *Betaarterivirus suid 1* and *Betaarterivirus suid 2*, respectively (Brinton et al., 2021; Kuhn et al., 2016).

The PRRSV genome is about 15 kb in length. It contains 5’- and 3’-untranslated regions (UTRs) flanking 11 known open reading frames. The 3’ region of the genome encodes four membrane-associated glycoproteins (GP2a, GP3, GP4 and GP5), three unglycosylated membrane proteins (E, ORF5a and M) and a nucleocapsid protein (N) (Fang and Snijder, 2010; Snijder et al., 2013). The replicase-associated genes, ORF1a and ORF1b, are situated in the 5’ region and represent nearly 75% of the viral genome. ORF1a and ORF1b encode two long nonstructural polyproteins, pp1a and pp1ab. The expression of ORF1b depends on a -1 ribosomal frameshift signal in the short region where ORF1a and ORF1b overlap. After translation, the pp1a and pp1ab polyproteins are proteolytically processed into at least 14 nonstructural proteins (nsps). The proteolytic cascade depends on four proteinase domains encoded in ORF1a, namely two papain-like proteases (PLP1α and PLP1β) located in nsp1α and nsp1β, a papain-like protease (PLP2) domain located at the N-terminal end of nsp2, and a serine protease (SP) located in nsp4. PLP1α autocleaves between nsp1α and nsp1β, PLP1β autocleaves between nsp1β and nsp2, and PLP2 cleaves between nsp2 and nsp3, together mediating the rapid release of nsp1α, nsp1β and nsp2 from the polyproteins (Li et al., 2012). The largest cleavage product of the replicase polyprotein is nsp2, which contains multiple domains for multiple functions (Fang and Snijder, 2010). Besides cleaving between nsp2 and nsp3, the PLP2 domain functions as a cofactor for the nsp4 serine protease during proteolytic processing of the C-terminal regions of pp1a and pp1ab (Li et al., 2012). In our previous study, a new ORF (TF) was identified in the nsp2 region (Fang et al., 2012). The TF ORF is expressed by a -2 programmed ribosomal frameshift (PRF) mechanism, which results in a transframe fusion protein (nsp2TF) that consists of the N-terminal two thirds of nsp2 joined to a unique C-terminal domain specified by the TF ORF. The same frameshift site was also found to direct an efficient -1 PRF, which generates a second nsp2 variant, named nsp2N (Fang et al., 2012; Snijder et al., 2013). In both PRRSV prototypic strains, Lelystad virus (PRRSV-1) and VR2332 (PRRSV-2), ribosomes making a -1 PRF immediately encounter a stop codon, thus yielding a truncated nsp2 with zero amino acids encoded by the -1 reading frame. Remarkably, frameshifting at the nsp2TF/nsp2N site requires the viral protein nsp1β and host poly(C) binding protein (PCBP) as transactivators (Li et al., 2014).

PRRS was initially recognized in the United States in the late 1980s, then in Europe during the early 1990s (Hill, 1990; Hopper et al., 1992; Plana et al., 1992; Wensvoort et al., 1991). Since then, PRRSV has rapidly evolved into one of the leading pathogens threatening the global swine industry. It causes numerous acute respiratory disease outbreaks and abortion storms (Albina, 1997; An et al., 2011; Done et al., 1996). More recently, highly virulent PRRSV-2 strains have emerged in US swine farms, including the strains characterized as 1-7-4 and 1-8-4 cutting pattern based on an ORF5 restriction fragment length polymorphism (RFLP) analysis (Han et al., 2006b; van Geelen et al., 2018). Since the fall of 2020, the presence of a highly pathogenic PRRSV-2 lineage 1C (L1C) strain has been reported by veterinarians from the Midwest region of the United States (Kikuti et al., 2021; Trevisan et al., 2021a; Trevisan et al., 2021b). A high percentage of nursery pigs have presented with clinical signs of thumping and coughing with increased mortality. Based on ORF5 sequence analysis, most of the viruses associated with these cases had RFLP 1-4-4 pattern and formed a separate subcluster within the sublineage L1C, for which reason these PRRS viruses have been referred to as “PRRSV 1-4-4 L1C variant” strains (Kikuti et al., 2021; Trevisan et al., 2021a). In this study, we performed a fundamental genetic characterization on a PRRSV RFLP 1-4-4 L1C variant field isolate. The unique -1 PRF signal sequence patterns that lead to the expression of a C-terminally extended nsp2N protein were analyzed in detail. The potential correlation of the PRF signal sequence changes with the growth ability of the emerging PRRSV variants was further explored. This study provides an insight into the genetic features that contribute to the emergence of PRRSV variants in the swine population.

## 2 Results

### 2.1 Isolation and phylogenetic analysis of an emerging PRRSV 1-4-4 L1C variant

In July of 2021, a serum sample was obtained from a swine farm located at Minnesota State in the US, in which nursery pigs had experienced a PRRSV outbreak. A PRRSV strain (UIL21-0712) was isolated by inoculating the swine serum onto a cell culture of porcine alveolar macrophages. Cytopathic effect of the cells was observed by 48 hours post inoculation.

**Figure 1:**
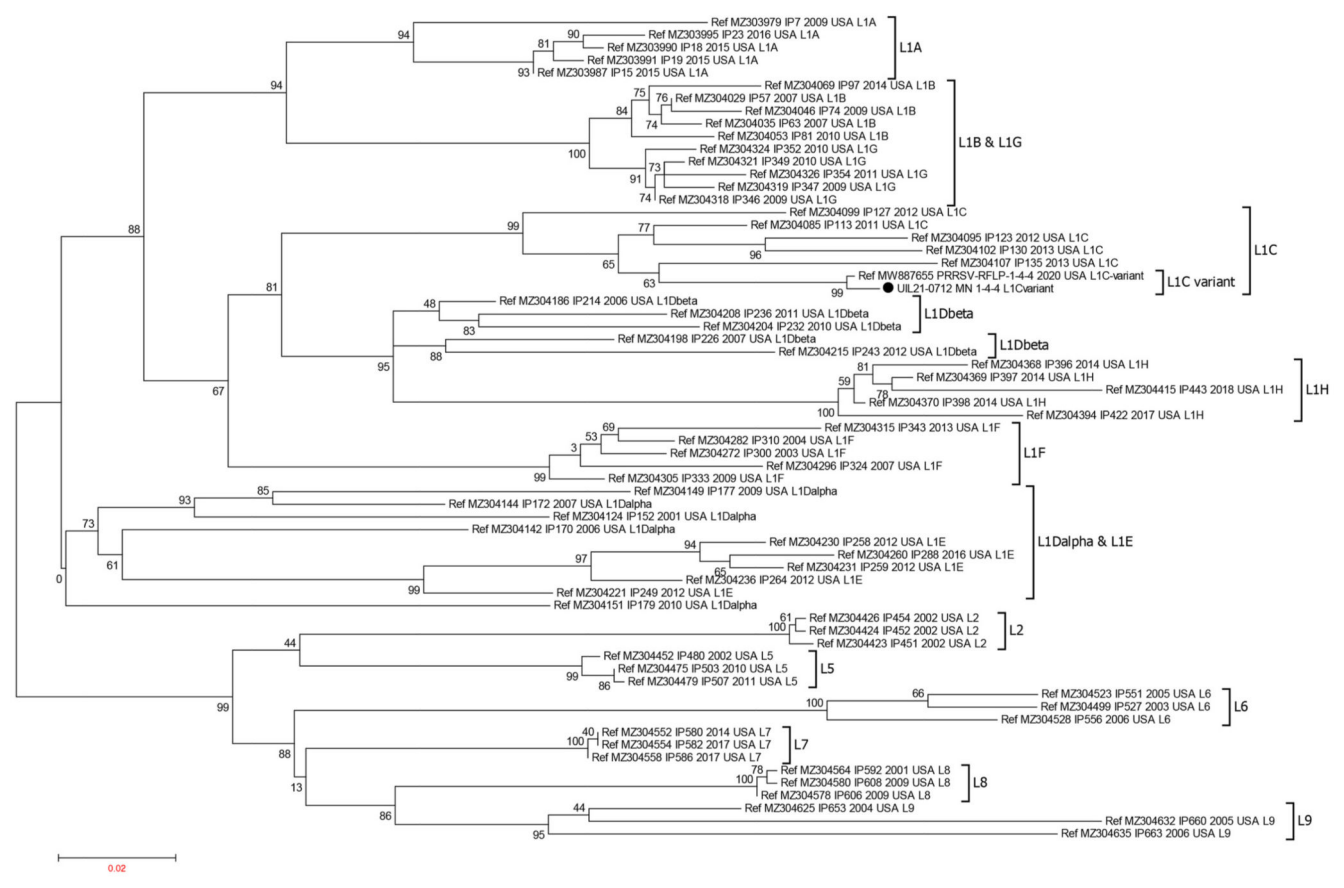
Phylogenetic analysis of PRRSV isolate UIL21-0712. The ORF5 nucleotide sequence of the PRRSV 1-4-4 L1C variant isolate UIL21-0712 together with reference sequences representing different lineages and sublineages of PRRSV-2 were used to construct the phylogenetic tree. The IQ tree was opened and annotated in MEGA6 software. Bootstrap analysis was carried out on 1000 replicates. The lineages and sublineages are indicated in the tree. The isolate UIL21-0712 is denoted by solid circle.

A full-length genome sequence was obtained from the first passage of the virus in the macrophages. Restriction fragment length polymorphism analysis based on ORF5 sequence showed that UIL21-0712 has an RFLP cutting pattern of 1-4-4. Phylogenetic analysis (Figure 1) of ORF5 sequences indicated that UIL21-0712 belongs to PRRSV-2 lineage 1C (L1C) (Paploski et al., 2021; Paploski et al., 2019) and is closely related to the PRRSV L1C variant strain (GenBank accession number MW887655) identified by Iowa State University in December 2020 (Trevisan et al., 2021a).

### 2.2 Genomic sequence analysis of PRRSV 1-4-4 L1C variant UIL21-0712

**Figure 2:**
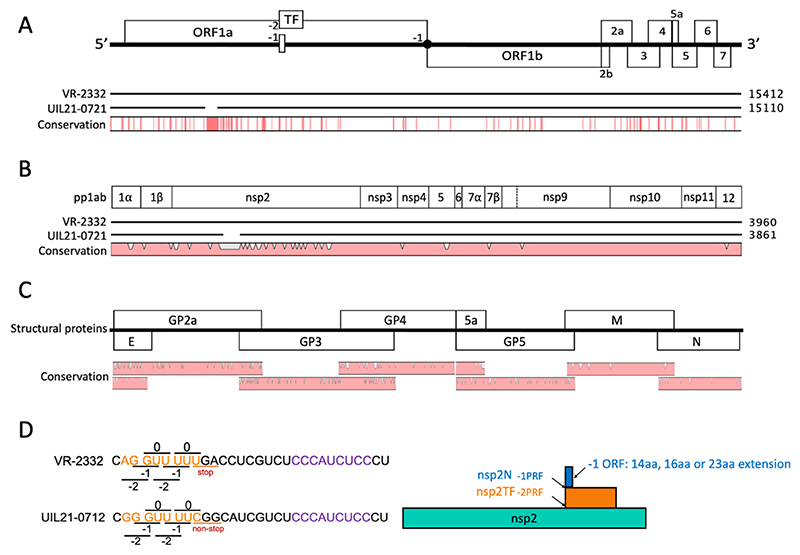
Full-length genome sequence comparison between PRRSV isolate UIL21-0712 and the prototype strain VR-2332. (**A**) PRRSV genome organization and genome nucleotide differences between UIL21-0712 and VR-2332. Programmed ribosomal frameshifts in the nsp2-coding region and the ORF1a/1b junction are indicated with −1 and −2. Depending on the viral isolate, the −1 frameshift in the nsp2-coding region is immediately followed by a stop codon (i.e. VR2332) or results in a small C-terminal extension of nsp2N (i.e. UIL21-0721). (**B**) Schematic representation of the amino acid differences between UIL21-0712 and VR-2332 within the ORF 1ab polyprotein. (**C**) Schematic representation of the amino acid differences between UIL21-0712 and VR-2332 within the structural proteins. (**D**) Sequence differences of -2/-1 PRF signal between UIL21-0712 and VR-2332 and a graphical representation of the nsp2N extension. Nucleotide sequence of the frameshift site is indicated in orange and the downstream C-rich motif is indicated in purple. “stop”, stop codon UGA; “non-stop”, substitution of “U” to “C” makes sense codon CGG.

The genome of UIL21-0712 contains 15,110 nucleotides (nt), excluding the poly(A) tail. In comparison with the prototypic PRRSV-2 strain, VR-2332, the full-length genomic sequence of UIL21-0712 showed 85.3% nt identity (Figure 2A; Table 1). The global pattern of nucleotide and amino acid differences between the two strains were further analyzed in detail. The identity varies for individual ORFs: ORF 1a is 79.5% nt identical with a 300-nt deletion, ORF 1b is 86.5% nt identical, and the ORF 2 to 7 region is 86.7% nt identical to the corresponding regions of VR-2332. The major differences cluster near the 5’ end and in the genomic regions coding for structural proteins GP2a, E and GP3-5 (Figure 2A).

The predicted proteins for most of ORFs 1a and 1b as well as ORFs 5a, 6, and 7 displayed greater than 90% amino acid (aa) identity with those of VR2332. However, the protein sequences of nsp1β, nsp2-related proteins (nsp2, nsp2TF and nsp2N), GP2a, E, and GP3-5 displayed less than 90% aa identity with those of VR-2332. The functional significance of the peptides containing the specific changes was not reported previously. The three nsp2-related proteins displaying the lowest degree of conservation at 54.6-64.6% aa identity. These regions were further studied in more detail (see below). A graphical representation of all amino acid differences observed between UIL21-0712 and VR-2332 is shown in Fig. 2B (ORF1ab), Fig. 2C (ORFs 2 to 7), and Fig. 2D (frameshifting signal).

### 2.3 Variations in the nsp2 region and new features of the -2/-1 PRF signal sequence

The nsp2 is the largest protein of PRRSV, with 1196 aa in VR-2332. The N-terminal PLP2 domain region is relatively conserved, whereas the central region of nsp2 is the most variable. Here, the UIL21-0712 genome contains 300-nt deletion (genome positions 2323 to 2622 nt of VR-2332; Figure 2A), resulting in a 100-aa deletion corresponding to aa positions 328–427 of VR-2332 nsp2. Therefore, the UIL21-0712 viral genome encodes a nsp2 protein of 1096 aa, which has 64.6% aa identity with the nsp2 of VR-2332. Since the N-terminal two thirds of the nsp2 sequence is identical to the zero-frame encoded portions of nsp2TF and nsp2N, both nsp2TF and nsp2N of UIL21-0712 contain similar deletion and insertion patterns compared to VR-2332, with aa identity levels of 58.8% and 54.6% aa, respectively (Table 1).

Further in-depth sequence analysis revealed that the sequence of -2/-1 PRF signal located within the nsp2 of UIL21-0712 differs from that of VR-2332 and other traditional PRRSV strains (Figure 2D). As we described previously (Fang et al., 2012), at the traditional slippery sequence (G_GUU_UU**U**) site, the -2 PRF generates the frameshifting product nsp2TF, while ribosomes that undergo a -1 PRF immediately encounter a stop codon (**UGA, UAG** or **UAA**), which terminates translation of the -1 reading frame to produce nsp2N. The UIL21-0712 isolate contains the slippery sequence G_GUU_UU**C**, and the -2 PRF generates a 919-aa nsp2TF protein. Remarkably, substitutions immediately downstream of the slippery sequence disrupt the -1 PRF stop codon (**UGA** to **CGG**; Figure 2D) in the genome of the UIL21-0712 isolate. This change extends the translation of nsp2N with an additional 23 aa C-terminal peptide (nsp2N+23aa).

**Figure 3:**
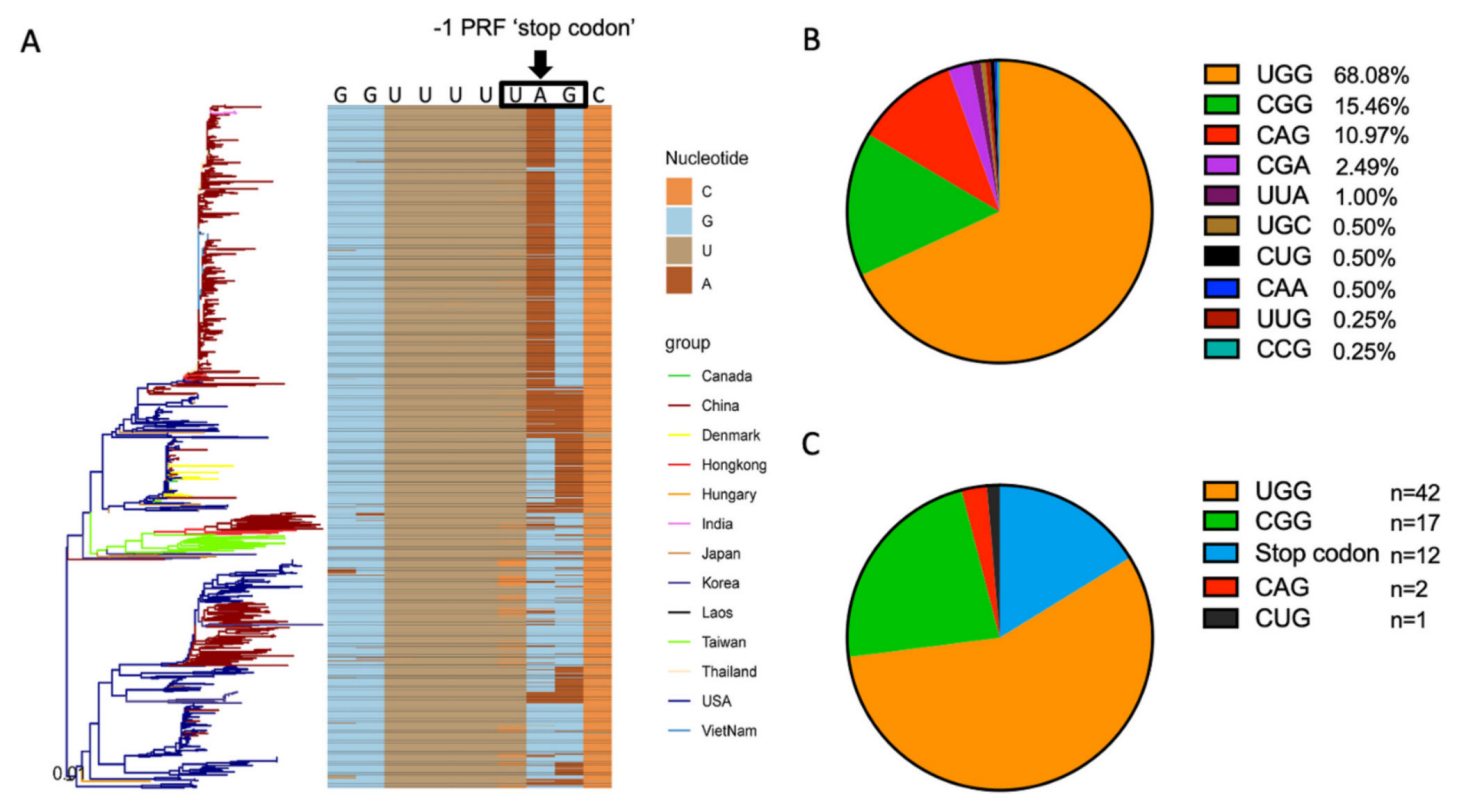
Sequence analysis of -1/-2 PRF signal region from PRRSV sequences in GenBank database and field isolates. (**A**) Phylogenetic analysis of PRRSV -2/-1 PRF variants. The frequency of the appearance of -2/-1 PRF variant patterns with their geographic location was analyzed using the available full-length nsp2 sequences in GenBank. The -2/-1 PRF signal regions were aligned using MAFFT (version 7.029) and MEGA X software. Phylogenetic tree was visualized and annotated with ggtree package. (**B**) Graphical representation of the frequency of different PRRSV -2/-1 PRF variant patterns in GenBank (as of Mar 15^th^, 2022). PRRSV isolates with traditional -1 PRF stop codons are not shown in the graph. (**C**) Analysis of recent field isolates for substitution patterns in the -1 PRF stop codon. A total of 74 selected field isolates from 2015 to 2021 provided by Iowa State University Veterinary Diagnostic Laboratory were sequenced for the -2/-1 PRF region in nsp2, and the identity of the codon at the traditional - 1 PRF stop codon position was determined.

The emergence of -1 PRF stop codon variants was traced back in the PRRSV sequences published in GenBank. Before 2011, in the majority of PRRSV full-length genome sequences (483/518), a -1 PRF would result in immediate termination at a −1 frame stop codon (with G_GUU_UU**U**_**ga**, G_GUU_UU**U**_**ag**, and G_GUU_UU**U**_**aa** found in different PRRSV isolates; −1 frame stop codons indicated in bold). However, the percentage of the stop codon variants has quickly increased after 2011 (with up to 50% of the sequences from 2011-2021 lacking a stop codon at this position; Figure 3B; Table 2). We further analyzed the -2/-1 PRF signal sequence region of 74 PRRSV isolates that were isolated from diagnostic samples submitted to the ISU Veterinary Diagnostic Laboratory during 2015 to 2021 (Figure S1, Table S1). Sequencing result revealed that 83.8% (62 of 74) of the isolates contain substitutions that disrupt the -1 PRF stop codon (Figure 3C). The changes in the -1 PRF stop codon sequence can be summarized into two major types among the emerging variants. In the first type of change, such as that in the UIL21-0712 isolate, the first nt of the stop codon, which is also the last nt of slippery sequence, is substituted from U to C. In the second type of change, such as that in the ISU20-32315 isolate, the first nt of the stop codon remains unchanged while substitutions occur in the last 1 or 2 nucleotides. These changes extend translation of the -1 PRF product, resulting in a unique 14, 16, 18 or 23-aa C-terminal peptide for nsp2N, except for two isolates with 39-aa extension and two isolates with an 87-aa extension (Table 2). Ten different -1 PRF stop codon variant patterns were identified using the GenBank database (Table 2). Two of the variant patterns (UGG and CGG) are predominantly circulating in the field (Figure 3). More importantly, these dominant variants were obtained from swine farms experiencing PRRSV outbreaks with increased mortality/morbidity, including the PRRSV RFLP 1-4-4 lineage 1C variants (Table S1).

### 2.4 Growth ability of recombinant viruses with different -1 PRF “stop codon” substitutions

**Figure 4:**
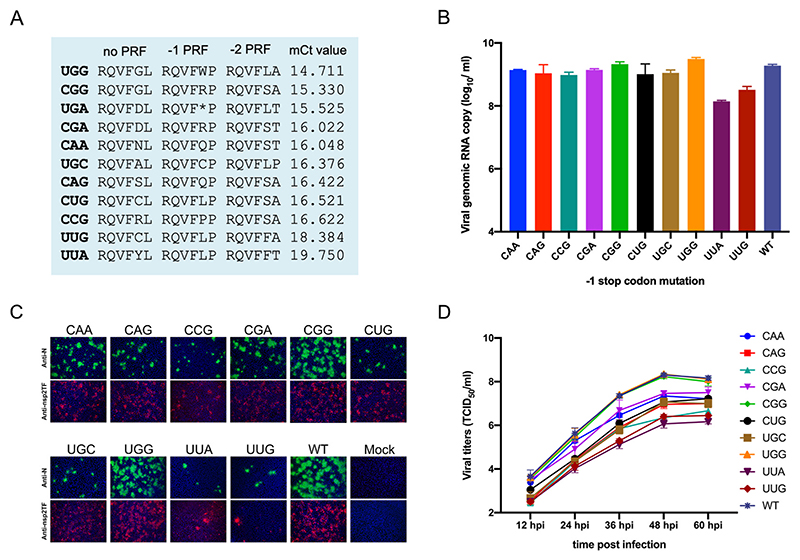
*G*rowth characterization of -2/-1 PRF mutants in cell culture. Recombinant viruses containing different -1 PRF “stop codon” variants were constructed by introducing mutations in PRRSV-2 infectious clone pSD95-21. (**A**) Amino acid sequences corresponding to the specific stop codon mutations introduced in pSD95-21 following no PRF, -1 PRF or -2 PRF on the G_GUU_UU[U/C] shift site. The qRT-PCR results (Ct value) for the wild type virus and -1 PRF mutants recovered from transfected BHK-21 cells are presented on the right end of the panel. (**B**) Viral RNA copy numbers of wild type virus or -1 PRF mutants from transfected cells. BHK-21 cells were transfected with equal amounts of full-length cDNA construct of WT or -1 PRF mutants. Cell lysates were harvested at 48 hours post transfection. Total RNA was extracted and viral RNA level was determined by quantitative real-time qRT-PCR. (**C**) Immunofluorescence assay detection of PRRSV nsp2TF and N protein expression in cells infected with different -1 PRF mutants. MARC-145 cells were infected with the wild type virus or a mutant at an MOI of 0.5 and fixed at 18 hours post infection. Cells were fixed and stained with DAPI (blue) and anti-N mAb (green) or anti-nsp2TF pAb (red). (**D**) Growth kinetics of wild type and -1 PRF mutants in cell culture. MARC-145 cells were infected with the wild type virus or a mutant at an MOI of 0.01, and cultural supernatants were harvested every 12 hours to measure the viral titer. WT, wild type virus; hpi, hours post infection.

To determine whether variations at the traditional site of the -1 PRF stop codon affect the viral growth ability, we constructed a panel of 10 recombinant viruses using a full-length cDNA infectious clone of PRRSV-2 isolate SD95-21, which contains the same traditional slippery sequence (G_GUU_UU**U_ga**) as that of VR2332. In each construct, a representative variant pattern was introduced (Table 2; Figure 4A). To rescue the recombinant viruses, this panel of constructs was initially transfected into BHK-21 cells and cell cultural supernatant (P0 virus) was harvested and passed on MARC-145 cells. For P0 viruses, the viral titer was measured by qRT-PCR. As shown in Figure 4A-B, the two mutants with each containing a dominant variant pattern (UGG or CGG) produced higher copy numbers of viral genome than that of the other mutants (Table S2.1-S2.2). Immunofluorescence assay (IFA) showed that PRRSV nsp2TF and N protein were detected in infected MARC-145 cells, indicating that viable recombinant viruses were recovered from the cell culture (Figure 4C). The growth kinetics of these PRF mutants and the parental virus SD95-21 were further compared. MARC-145 cells were infected with each virus (same genome copy number as measured by qRT-PCR) and the culture supernatant was harvested at 12, 24, 36, 48 and 60 hpi and viral titers were determined by TCID_50_. The result showed that the two mutants with the dominant variant pattern (UGG or CGG) exhibit similar growth kinetics as the parental virus SD95-21 (Figure 4D; Table S3.1-S3.2). The parental virus, UGG and CGG mutants all reached a peak titer of about 10^8^ TCID_50_/ml at 48 hpi. The rest of the mutants showed varying degrees of reduced growth kinetics, with the UUG and UUA mutants exhibiting the greatest reduction in peak titer (2-log reduction compared to that of the parental virus SD95-21; Table S3.1-S3.2).

### 2.5 Effect of -1 PRF stop codon variation on the frameshifting efficiency

**Figure 5:**
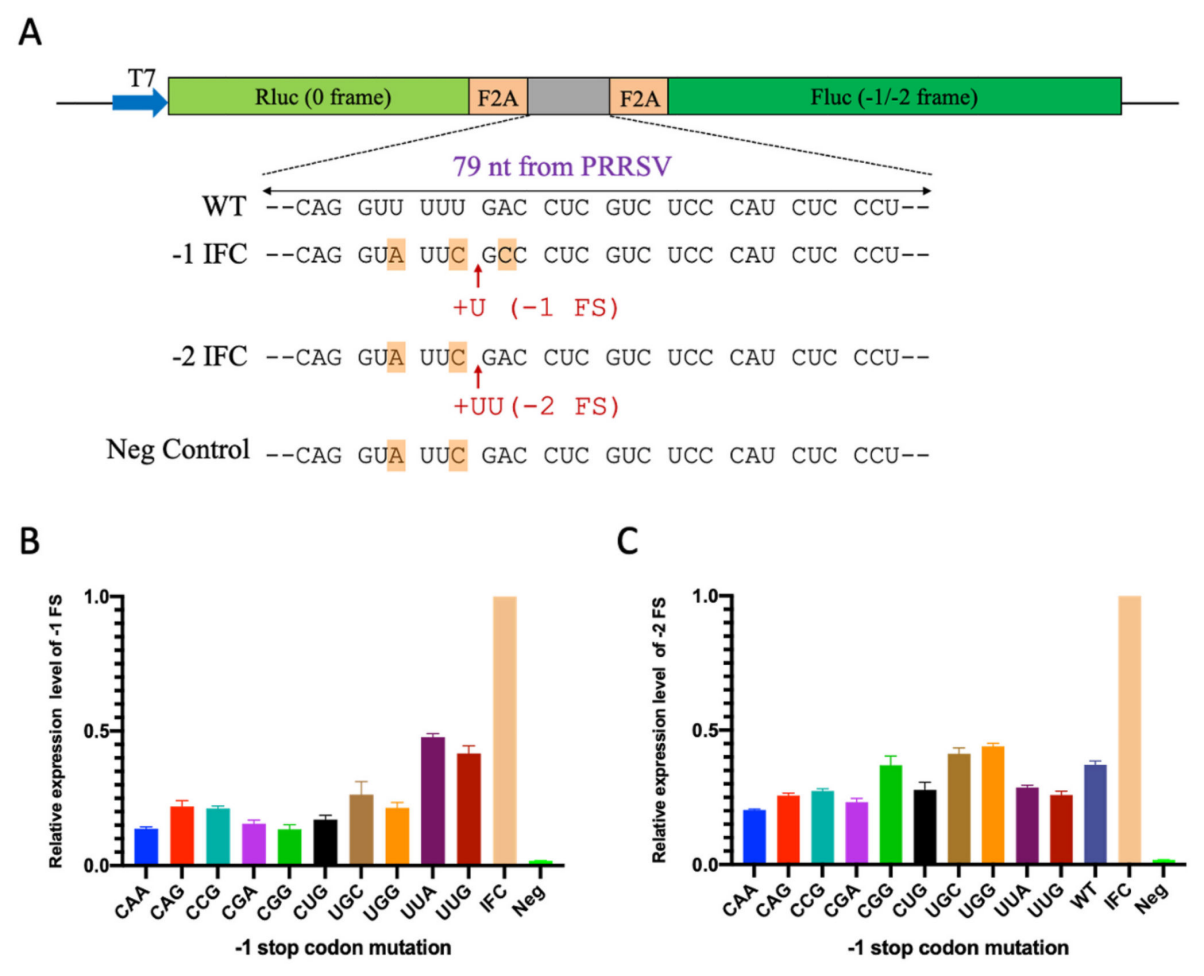
Dual luciferase reporter assay measuring the efficiency of -2/-1 PRF for different -1 PRF stop codon variants. (**A**) Schematic representation of the dual luciferase constructs. A 79-nt sequence containing the PRRSV -2/-1 PRF signal was inserted between *Renilla* luciferase (Rluc) and firefly luciferase (Fluc) ORFs so that Fluc is in the frameshift (FS) frame downstream of Rluc. The in-frame control (IFC) constructs were generated by inserting one U (-1FS) or two Us (-2 FS) after the slippery sequence and mutating the slippery site from G_GUU_UUU to G_GUA_UUC. For the -1 FS IFC construct, an “A” downstream of the slippery sequence was also mutated to “C” to avoid the termination of translation by the stop codon UGA. The negative control (Neg) constructs were generated by simply mutating the slippery site from G_GUU_UUU to G_GUA_UUC. HEK-293T cells were co-transfected with different luciferase constructs and a plasmid DNA encoding PRRSV nsp1β at a ratio of 4:1. At 24 hours post transfection, cell lysates were harvested to measure the luciferase expression levels. (**B**) -1 PRF efficiency of different stop codon mutants. (**C**) -2 PRF efficiency of wild type or different stop codon mutants. The PRF efficiency for each mutant was calculated by the relative value of their Fluc/Rluc ratio to the Fluc/Rluc ratio of the IFC. WT, wild type. FS, frameshifting.

As previously described (Fang et al., 2012), PRRSV replication is highly regulated with an optimized expression ratios for the different proteins. In prototypic PRRSV strains, frameshifting efficiencies at the nsp2 PRF signal were measured to be in the range of 23-39% and 6–7% for −2 PRF and −1 PRF, respectively (Cook et al., 2022; Fang et al., 2012). To determine whether substitutions in the -1 PRF stop codon site affects the frameshifting efficiency, we used a previously described strategy to generate two sets of reporter gene constructs (Li et al., 2014). In all the constructs, PRRSV RNA sequences from the PRF-inducing region (the slippery sequence and downstream C-rich region) were placed between two luciferase genes [pSGDLuc; (Loughran et al., 2017); Fig. 5A], and the ORF1a frame of the PRF sequence was placed in-frame with the upstream (*Renilla*) luciferase gene. For the set of -1 PRF constructs, the downstream (firefly) luciferase was placed in the -1 frame so that its expression depends on the occurrence of -1 PRF; and for the set of -2 PRF constructs, the firefly luciferase was placed in the -2 frame to measure the efficiency of -2 PRF. In each construct, the stop codon (UGA) on the traditional slippery sequence (GGUUUU**Uga**) was mutated into one of the stop codon variants as listed in Table 2. As controls, an in-frame control (IFC) construct was constructed, in which the *Renilla* and firefly luciferase genes were placed in the same frame with the insertion of one nucleotide (T in -1 PRF constructs) or two nucleotides (TT in -2 PRF constructs) immediately downstream of the slippery sequence. A negative control (shift site mutant) was also constructed, in which the frameshift site GGUUUUU is mutated to GGUAUUC. Frameshifting efficiencies were determined by comparing the ratio of enzymatic activities of firefly and *Renilla* luciferase in parallel HEK-293T cell cultures transfected with individual pSGDLuc test constructs, normalized by the same ratio for the in-frame control construct. In all cases, constructs were co-transfected with a plasmid expressing nsp1β, the viral essential transactivator of PRF at the nsp2 PRF site.

As shown in Fig. 5B, for the panel of -1 PRF constructs, the UUA and UUG variants that had the lowest growth kinetics showed substantially higher levels of -1 PRF efficiency compared to the other variants (*p*-value = 0.00044; see Methods). Compared to the IFC control, the -1 PRF efficiency for the UUA and UUG variants is 47.7% and 41.6%, respectively, while the rest of the variants showed -1 PRF efficiencies in the range 13.5-26.4%. For the panel of -2 PRF constructs (Fig. 5C), the UGC variant and two growth dominant variants UGG and CGG, in addition to WT, showed relatively higher levels of -2 PRF (41.2%, 44.0%, 37.0% and 37.2%, respectively) compared to the other variants (*p*-value = 0.00021; see Methods), which had -2 PRF efficiencies in the range 20.2-28.6%.

## 3 Discussion

In this study, the PRRSV isolate UIL21-0712 was obtained in a serum sample from a swine farm in Minnesota, where a PRRSV outbreak was reported in Spring 2021. Sequence analysis revealed that UIL21-0712 has a RFLP cutting pattern of 1-4-4. It belongs to a distinct phylogenetic clade of PRRSV-2 lineage 1C, which includes other emerging isolates that were reported to cause severe clinical manifestations in infected pigs during 2020-2021(Kikuti et al., 2021; Trevisan et al., 2021a) indicating a shared origin for these isolates. These emerging isolates have been referred to as L1C 1-4-4 variant strain (Kikuti et al., 2021; Pamornchainavakul et al., 2022; Trevisan et al., 2021a). Analysis of 19 L1C 1-4-4 whole genome sequences (WGS) and 232 published PRRSV-2 WGS collected during 1995-2021 suggest that the recently emerged L1C variant descended from a recombinant ancestor involving recombination at the ORF1a gene between two viruses that were classified as L1C and L1A based on ORF5 sequences (Pamornchainavakul et al., 2022).

The genome of UIL21-0712 shares 99%-99.51% identity with the other reported 1-4-4 L1C variant isolates, but only shares 82.45% nt identity with the PRRSV-2 prototypic strain VR2332. Considering the rapid evolution of RNA viruses and the 28-year interval between the identification of VR2332 and the 1-4-4 L1C variant strain, such large genetic differences may be expected. Although the origin of the 1-4-4 L1C variant strain is unclear, genome sequence comparison to VR2332 suggests certain regions of the viral genome may be more tolerant to mutations than others, and thus may evolve more rapidly. These regions include nsp1β, nsp2, and ORFs 2-5. Nsp2 encodes the largest viral protein. In fact, the N-terminal PLP2 domain of nsp2 (and nsp2TF and nsp2N), which is important in proteolytic processing of the viral replicase (Han et al., 2009), is well-conserved. The central region of nsp2 has been reported to be the most variable part of the genome with deletions found in various strains (Fang et al., 2004; Gao et al., 2004; Tian et al., 2007; van Geelen et al., 2018; Yu et al., 2020). The nsp2 sequence of UIL21-0712 is consistent with that notion, containing a 100-aa deletion, which is also found in the other 1-4-4 L1C variant field isolates. In previous studies, nsp2 deletions have been suspected to relate to increased virulence. However, further studies showed that, although nsp2 deletions are a key characteristic of some emerging strains, there is no direct evidence to correlate the deletions with increased virulence (van Geelen et al., 2018; Zhou et al., 2009). One assumption is that the flexibility of this region could be caused by immunological pressure, as a panel of B- and T-cell epitopes were identified previously (Chen et al., 2010; Oleksiewicz et al., 2001). As the virus continually evolves, it may eliminate the genomic region that is under host immune pressure for survival (Han et al., 2006a; Yoshii et al., 2008). The generation of the nsp2TF and nsp2N proteins through -2/-1 PRF in the nsp2 region complicates this situation. As nsp2TF and nsp2N contain sequence identical to the N-terminal two thirds of nsp2, the 100-aa deletion is also present in both these proteins. Both nsp2TF and nsp2N were determined to function as innate immune antagonists to suppress host innate immune responses (Guo et al., 2021; Li et al., 2018), and nsp2TF was also demonstrated to interact with major viral envelope proteins to promote viral replication (Guo et al., 2021). Whether these deletions affect the functions of nsp2TF and nsp2N warrants further investigations.

Our in-depth sequence analysis showed that changes identified in the PRF signal sequence in the nsp2 region disrupt the -1 PRF stop codon thus extending the translation of nsp2N with an additional 14-87 aa C-terminal peptide. Based on our analysis with the PRRSV sequences in GenBank, the proportion of sequences containing stop codon substitutions has increased more than seven-fold since 2011. This is consistent with the sequence analysis results from the 74 field isolates obtained by the ISU veterinary diagnostic laboratory during 2015-2021. Ten different stop codon variant patterns were found. Analysis of the sequences in GenBank and field isolates consistently showed that the UGG or CGG variants are dominant (83.7% of analyzed isolates) in the field, while the other variants, including UUA and UUG, have a lower frequency of appearance. Our reverse genetic study suggested a possible factor underlying these differences in frequency. In analysis of the growth ability of recombinant viruses with stop codon mutations, we observed differences in viral growth between the different mutants. Remarkably, the UGG and CGG mutants that are dominant in the field grew to higher viral titers than the other mutants, while the UUA and UUG variants had the lowest viral titers. Compared to the parental virus, the UGG and CGG mutants do not show significant difference in viral growth ability (albeit these experiments were in the context of the VR2332 backbone). Thus the evolutionary path from stop codon variants to CGG/UGG variants may - potentially - be one of neutral genetic drift at this site. Nonetheless, it is interesting that traditional variants predominantly have a stop codon here (with UGA, UAA and UGA all represented - suggesting that there was an advantage to having a stop codon in the context of those traditional viral genomes), whereas newer emerging variants often replace the stop codon with one of various sense codons, perhaps facilitated by changes nearby or elsewhere in the genome.

Virus replication is known to be highly regulated with an optimized ratio for different protein products (Li et al., 2014). For PRRSV and most arteriviruses, the balance between the synthesis of the pp1a and pp1ab replicase polyproteins is regulated by two ribosomal frameshift events, the -2/-1 PRF in the nsp2 region, and the −1 PRF at the ORF1a/1b junction. This leads to a complex series of ratios. Based on our previous analysis of typical PRRSV strains, of the ribosomes that translate nsp1α/nsp1β, approximately 20% synthesize nsp2TF, 7% synthesize nsp2N, and the other 73% synthesize nsp2–8, with only about 15% of ribosomes translating the ORF1b-encoded proteins (nsp9–12). In our recent ribosome profiling work, these PRF efficiencies have also been shown to vary over the time course of infection (Cook et al., 2022). Our luciferase assay results suggest that some -1 PRF stop codon changes may affect the efficiency of −2/−1 PRF. In general, the G_GUU_UUU to G_GUU_UUC substitution may be expected to reduce both -1 and -2 PRF since UUU and UUC are both decoded by the same phenylalanine tRNA isoacceptor whose anticodon, 3’-AAG-5’ has a higher affinity for UUC than for UUU (Eisinger et al., 1971). Consistent with this hypothesis, the CNN -1 stop codon mutations generally correlated with lower -1 and -2 PRF efficiencies than the UNN -1 stop codon mutations (Figures 5B and C).

Intriguingly, the -1 PRF efficiency was particularly enhanced when the traditional -1 frame stop codon was substituted with UUA or UUG. These variants had approximately 2-fold higher -1 PRF efficiencies than the UGC and UGG variants but lower -2 PRF efficiencies. One possible explanation is that UUA and UUG, but not UGC and UGG, could allow two consecutive -2 PRFs. Ribosomes which shift -2 nt (P and A sites moving from GUU_UUU to AGG_UUU) and then translocate one codon (P and A sites moving from AGG_UUU to UUU_UUU, where the two underlined ‘U’s correspond to the UU of the UUA or UUG codons), may then be able to undergo a second -2 shift (P and A sites moving from UUU_UUU to GGU_UUU). Since this second -2 shift would begin when the ribosome is 1 nt further 3’ than the normal -2 PRF, the spacer to the CCCANCUCC nsp1β/PCBP binding site is 1 nt shorter, which would be expected to enhance the level of -2 slippage relative to -1 slippage (Napthine et al., 2016). Thus, a second slip will increase the proportion of ribosomes entering the -1 frame and decrease the proportion of ribosomes entering the -2 frame. In contrast, for the UGC and UGG mutants, following a -2 slip and one codon translocation, the P and A sites would be positioned on UUU_UUG, a sequence that is much less slip-prone than UUU_UUU (Brierley et al., 1992) and ribosomes would therefore be much less likely to engage in a second slip.

It is intriguing that the viral growth titer of -1 PRF stop codon mutants correlates with their frequency of appearance in the field. It still needs to be determined whether changes in viral titer are due to the C-terminal extension to nsp2N, altered amino acids in nsp2 and/or nsp2TF, or altered frameshifting efficiencies. One possibility is that too much nsp2N production may be detrimental to viral replication. Currently, the function of nsp2N in viral replication is unknown since in traditional PRRSV strains, the presence of the -1 frame stop codon immediately following the frameshift site means that the entire nsp2N aa sequence is shared with nsp2/nsp2TF and it has therefore been difficult to separate the function of nsp2N from nsp2/nsp2TF in the context of virus-infected cells. With the unique C-terminal peptide of nsp2N found in the -1 PRF stop codon variants, it becomes possible to develop methods to study the specific function of nsp2N in viral replication and pathogenesis. Furthermore, we cannot exclude the possibility that the altered amino acids in the PRF signal sequence region could modulate nsp2TF, nsp2 or nsp2N function. For example, comparing the amino acids present in the different mutants, the top four viruses in terms of viral titer (UGA [WT], UGG, CGG and CGA) have zero-frame sequences of RQVF**G**L or RQVF**D**L, whereas the other seven viruses have N, A, R, S, C or Y but not G or D at the position marked in bold. However, G and D are physicochemically very different; thus, it is unlikely that D or G is providing the advantage to these viruses. On the other hand, the mutants with the lowest titers, UUA and UUG, have -2 PRF sequences of RQVF**FT** or RQVF**FA**, where the T or A are also present in the top four viruses but the second F is unique to the UUA and UUG mutants (S or L in all other mutants and WT), so it is possible that F at this position is detrimental to nsp2TF function. Due to the multiple frames and functions encoded in the shift site sequence, however, it is difficult to disentangle these possibilities in the context of virus infection.

Nonetheless, as discussed above, the viral replication process is highly regulated with an optimized ratio for different protein products, and differences in the -1/-2 PRF efficiencies would also cause difference in the expression levels of downstream replicase subunits nsp3-nsp12 that are more likely to have phenotypic effects than single amino acid changes, which in turn affect the overall viral replication. Further studies are warranted to elucidate whether and how the changes in the PRF signal sequence relate to the virulence of emerging PRRSV strains.

## 4 Methods

### 4.1 Cells and viruses

BHK-21 and MARC-145 cells were cultured in Minimum Essential Medium (MEM) (Gibco, Carlsbad, CA) supplemented with 10% fetal bovine serum (Sigma Aldrich, St. Louis, MO), antibiotics [100 units/ml of penicillin (Gibco, Carlsbad, CA) and 100 ug/ml of streptomycin (Gibco, Carlsbad, CA)] and 0.25 ug/ml fungizone (Gibco, Carlsbad, CA) at 37 °C with 5% CO2. Primary porcine alveolar macrophages (PAM) were cultured in RPMI 1640 medium (Gibco, Carlsbad, CA) supplemented with 10% fetal bovine serum and antibiotics at 37°C with 5% CO2. Infected PAM cells were maintained in MEM supplemented with 2% horse serum (HyClone, Logan, UT) at 37°C with 5% CO2.

A PRRSV positive serum sample was obtained from a swine farm in the Midwest of the US, in which nursery pigs were experiencing respiratory diseases with increased mortality. The UIL21-0712 strain was isolated by inoculating the serum sample into the cell culture of PAMs as described previously (Ropp et al., 2004). PRRSV infection was confirmed by observation of cytopathic effect and indirect immunofluorescence assay (IFA) as described in previous studies (Ropp et al., 2004; Shang et al., 2017). Viruses (cell culture supernatants) were harvested between 24 and 48 hours post infection (hpi).

Seventy-four contemporary PRRSV-2 field isolates were obtained at the Iowa State University Veterinary Diagnostic Laboratory during 2015-2021 from the clinical cases that experienced PRRSV outbreaks. These 74 PRRSVs were isolated in either MARC-145 or ZMAC cells following the previously described procedures (Yim-Im et al., 2021).

### 4.2 Genome sequencing and sequence analysis

The passage 1 of the UIL21-0712 isolate from the PAM were subjected to Sanger sequencing by Genscript (Piscataway, NJ). The genome sequences were completed by GeneRacer (Invitrogen), and the full-length genome sequence was submitted to GenBank (accession No. ON157048). The genome sequence identity of UIL21-0712 was initially compared with that of prototypic PRRSV-2 strain VR-2332 (GenBank accession No. AY150564.1). Complete PRRSV genome sequences were further aligned with representative PRRSV genome sequences obtained from GenBank using the ClustalW algorithm in MEGA 7.0 software.

The ORF5 sequences of 74 ISU PRRSV-2 isolates were determined by the Sanger method following the previous described protocols (Zhang et al., 2017) and the sequences were deposited to GenBank with accession numbers ON053119-ON053192. The ORF5-based genetic lineages of these 74 isolates were determined using the genetic classification system described previously (Paploski et al., 2021; Paploski et al., 2019; Shi et al., 2010). The partial nsp2 sequences spanning the -2/-1 PRF region of these 74 isolates were also determined by the Sanger method at the DNA sequencing facility in University of Illinois at Urbana-Champaign. The detailed information of these 74 PRRSV isolates is provided in Table S1.

Phylogeny for ORF5 nucleotide sequences was inferred with the maximum likelihood algorithm using the best-fitting model with a gamma distribution. The topology of the phylogenetic tree was assessed with 1000 bootstrap replicates. For analysis of nsp2 nucleotide sequences, all available PRRSV sequences with a complete nsp2 sequences were downloaded from GenBank. Sequence alignment was performed using MAFFT (version 7.029) (Katoh and Standley, 2013), and truncated by MEGA X (Kumar et al., 2018) to cover the complete coding region of nsp2. The maximum likelihood tree was built using IQ-TREE (Nguyen et al., 2015). The distribution frequency for the -2/-1 PRF variant patterns with their geographic location was visualized by using ggtree package in R studio(Yu, 2020).

### 4.3 Construction and recovery of -1 PRF mutants

A panel of 10 recombinant viruses with -2/-1 PRF site mutations was constructed using a full-length cDNA infectious clone of PRRSV-2 isolate SD95-21 (Li et al., 2013). For constructing each mutant, the upstream and downstream regions of the -2/-1 PRF slippery site were amplified and assembled using NEBuilder® HiFi DNA Assembly Cloning Kit. The recombinant viruses were launched by transfecting BHK-21 cells as described previously (Li et al., 2013). Briefly, BHK-21 cells (70-80% confluency) were transfected with 500 ng of the full-length cDNA clone pSD95-21 or its mutants using TransIT^®^-LT1 Transfection Reagent (Mirus, Madison, WI). At 48 h post transfection, cell culture supernatant was harvested and passaged onto MARC-145 cells. The viability of recombinant viruses was confirmed by immunofluorescence assay using rabbit polyclonal antibody (anti-nsp2TF) and mAb SDOW17 (anti-N protein) as described previously (Fang et al., 2012).

### 4.4 Real-time qRT-PCR

BHK-21 cells were transfected with 500 ng of the full-length cDNA clone pSD95-21 or its mutants using TransIT^®^-LT1 Transfection Reagent (Mirus, Madison, WI). At 48 h post transfection, total RNA was extracted using SV Total RNA Isolation System (Promega, Madison, WI) from the cell lysates and PRRSV viral RNA copy number was determined by using EZ-PRRSV™ MPX 4.0 Master Mix and Enzyme kit (Tetracore, Rockville, MD).

### 4.5 Growth Kinetics

Growth kinetics of the wild-type and recombinant viruses were examined by infecting MARC-145 cells at an MOI of 0.01. Infected cells were collected at 12, 24, 36, 48, and 60 hours post-infection (hpi). Viral titers were determined by TCID_50_ as described previously (Li et al., 2013).

### 4.6 Dual luciferase assay

The dual luciferase vector pSGDluc (version 3) was a kind gift from John Atkins (Loughran et al., 2017). To construct plasmids for the dual-luciferase assay, a 79-nt oligonucleotide (nucleotides 3877–3955 of the PRRSV-2 SD95-21 genome) containing the wild-type sequence or mutations (Figure 5A) of the frameshifting signal was synthesized and cloned into pSGDluc. As controls, in-frame control (IFC) constructs were constructed, in which the *Renilla* and firefly luciferase genes were placed in the same frame with the insertion of one nucleotide (T in -1 PRF constructs) or two nucleotides (TT in -2 PRF constructs) immediately downstream of the slippery sequence. A negative control (shift site mutant) was also constructed, in which the frameshift site GGUUUUU was mutated to GGUAUUC. The plasmid for expression of PRRSV nsp1β (pFlag-nsp1β) was constructed by PCR amplification of the nsp1β-coding region (genome nucleotides 731 to 1339) of the PRRSV-2 SD95-21 strain, followed by cloning into the plasmid vector p3xflag-cmv-24 (MilliporeSigma, Rockville, MD). The luciferase assay was performed using TransIT^®^-LT1 Transfection Reagent (Mirus, Madison, WI) follow the manufacture’s instruction. HEK-293T cells were co-transfected with 0.4 μg pSGDluc containing the PRRSV PRF sequence and 0.1 ng pFlag-nsp1β. At 24 h post-transfection, cells were harvested, and luciferase expression was measured using the Dual-Luciferase^®^ Reporter Assay System (Promega, Madison, WI) and a luminometer (Perkin Elmer Victor2 Microplate Reader, Perkin Elmer, Waltham, MA). Frameshifting efficiencies were calculated as the ratio of firefly to *Renilla* luciferase activities for the test construct, divided by the same ratio for the corresponding IFC plasmid.

For the data in Figures 5B and 5C, to divide the tested constructs into “high” and “low” PRF efficiency groups in an unbiased manner, for each of Figures 5B and 5C, we listed the mean PRF efficiency values for the tested constructs in numerical order. We then considered all possible divisions of the list into “high” and “low” PRF efficiency subgroups (e.g. for Figure 5B, there are 10 tested constructs hence 9 possible divisions of the numerically ordered values into high and low subgroups). For each possible division, we compared the high and low subgroups using a *t*-test (one tailed, equal variances). We then selected the division with the lowest *p*-value as being the optimal division of the constructs into “high” and “low” PRF efficiency subgroups, and scaled the corresponding *p*-value by the number of tests performed (i.e. 9 for Figure 5B, 10 for Figure 5C) as a Bonferroni correction for multiple testing. This resulted in UUA and UUG forming the high PRF efficiency subgroup for Figure 5B (with corrected *p*-value 0.00044) and CGG, UGC, UGG and WT forming the high PRF efficiency subgroup for Figure 5C (with corrected *p*-value 0.00021).

## Supplementary Material

Figure S1 Phylogenetic analysis of ORF5 nucleotide sequences of 74 ISU PRRSV-2 isolates and the UIL21-0712 isolate included in this study together with reference sequences representing different lineages and sublineages of PRRSV-2. The IQ tree was opened and annotated in MEGA6 software. Bootstrap analysis was carried out on 1000 replicates. The lineages and sublineages are indicated in the tree. Among the 75 PRRSV-2 isolates included in this study, 42 isolates in which the traditional -1 PRF stop codon is altered to “UGG” are denoted by red triangles, 18 isolates with “CGG” are denoted by dark blue circles, 11 isolates with “UGA” are denoted by black diamonds, 1 isolate with “UAA” is denoted by a purple square, 2 isolates with “CAG” are denoted by green squares, and 1 isolate with “CUG” is denoted by a light blue square.

Table S1

Table S2

Table S3

## Figures and Tables

**Table 1:** Nucleotide and amino acid sequence comparison of UL21-0712 with VR-2332

Region	Bases	Nucleotide Length	Amino Acid Length	% Pairwise Identity Nucleotide/amino acid
**Full-length**	1-15110	15110	-	85.3/-
**5’ UTR**	1-188	188	-	93.1/-
**ORF1a**	189-7400	7,212	2404	79.5/78.3
**nsplα**	189-728	540	180	85.9/93.9
**nspiβ**	729-1337	609	203	80.6/78.3
**nsp2**	1338-4625	3288	1096	71.5/64.6
**nsp2N**	1338-3587, 3587-3658	2322	773	65.3/54.6
**nsp2TF**	1338-3587, 3586-4095	2760	919	69.0/58.8
**nsp3**	4626-5315	690	230	85.8/94.3
**nsp4**	5316-5927	612	204	91.8/94.6
**nsp5**	5928-6437	510	170	95.5/95.3
**nsp6**	6438-6485	48	16	93.8/100
**nsp7α**	6486-6932	447	149	82.1/92.0
**nsp7β**	6933-7262	330	110	82.4/83.6
**nsp8**	7263-7400	138	45	89.6/91.1
**ORF1b**	7397-11770	4374	1458	86.5/95.5
**nsp9**	7263-7394, 7394-9316	2055	685	87.8/96.1
**nsp10**	9317-10639	1323	441	85.7/95.0
**nspll**	10640-11308	669	223	85.8/94.6
**nsp12**	11309-11770	462	153	84.5/91.5
**ORF2a**	11772-12542	771	256	87.4/84.3
**ORF2b**	11777-11998	222	73	88.7/87.7
**ORF3**	12395-13159	765	254	83.8/83.1
**ORF4**	12940-13476	537	178	85.8/85.5
**ORF5a**	13477-13617	141	46	90.8/91.3
**ORF5**	13487-14089	603	200	86.4/85.1
**ORF6**	14074-14598	525	174	89.3/93.7
**ORF7**	14588-14959	372	123	89.5/92.7
**3’UTR**	14969-15110	151	-	92.1/-

**Table 2:** Summary of PRRSV -2/-1 PRF variation patterns and their frequency of occurrence before and after 2011.

	Before 2011	2011-2013	2014-2017	2018-2021	"-1 PRF"		"-2 PRF "	
Slippery sequence	No. of genomes	No. of genomes	No. of genomes	No. of genomes	length	No. of genomes	length	No. of genomes
**NN_NUU_UUU AAN**								
**NN_NUU_UUU AGN**	483	317	770	145	0	1715		
**NN_NUU_UUUGAN**								
**NN_NUU_UUU GCN**	0	1	1	0	23aa	2		
					14a	6		
					16aa	124		
**NN_NUU_UUU GGN**	28	23	145	77	18aa	10		
					23aa	130		
					39aa	2		
					87aa	1		
**NN_NUU_UUU UAN**	1	0	2	1	16aa	1		
					23aa	3	169aa	2116
**NN_NUU_UUU UGN**	0	0	2	0	16aa	1		
					23aa	1		
**NN_NUU_UUC AAN**	0	0	1	0	16aa	1		
					Maa	1		
**NN_NUU_UUC AGN**	3	19	21	1	16aa	34		
					23aa	9		
**NN_NUU_UUC CGN**	0	0	1	0	87aa	1		
**NN_NUU_UUC GAN**	0	7	3	0	16aa	5		
					23aa	5		
**NN_NUU_UUC GGN**	3	12	30	17	16aa	6		
					23aa	56		
**NN_NUU_UUC UGN**	0	0	1	1	16aa	2		
