# Supplementary figures and images for: Molecular characterization of emerging variants of PRRSV in the United States: new features of the -2/-1 programmed ribosomal frameshifting signal in the nsp2 region
Source: Virology. Author manuscript; Available in PMC 2026 Mar 10. (PMC7618843; doi:10.1016/j.virol.2022.06.004)

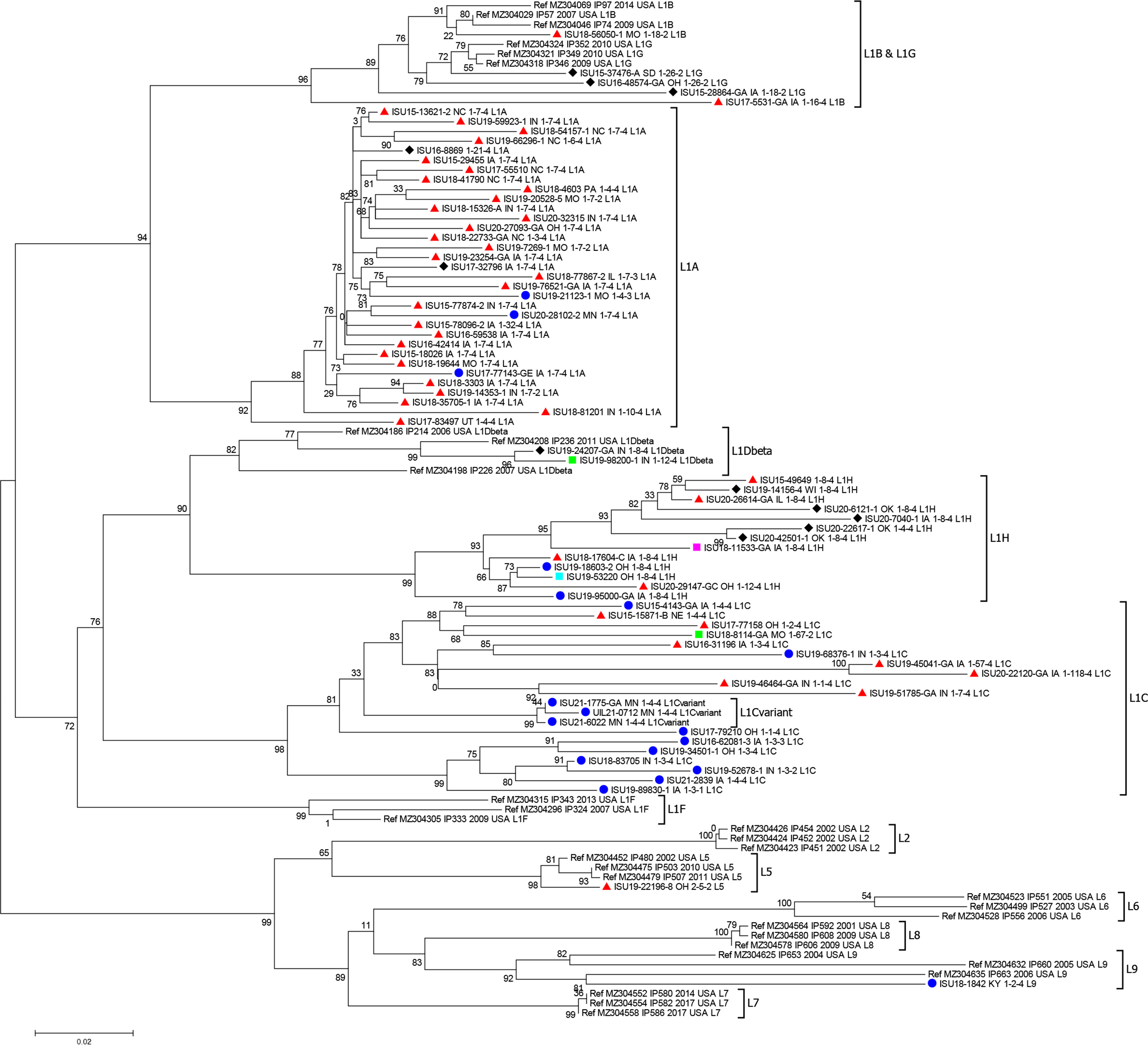

Supplement: Figure S1 [file EMS212717-supplement-Figure_S1.jpg]
