# Supplementary material for: Molecular characterization of emerging variants of PRRSV in the United States: new features of the -2/-1 programmed ribosomal frameshifting signal in the nsp2 region
Source: Virology. Author manuscript; Available in PMC 2026 Mar 10. (PMC7618843; doi:10.1016/j.virol.2022.06.004)
Supplement: Table S1 [file EMS212717-supplement-Table_S1.docx]

Table S1. Information of 74 PRRSV-2 isolates obtained during 2015-2021 from clinical cases experiencing PRRSV outbreaks

| **PRRSV-2**  **isolate name** | **Cells for virus isolation** | **Collection site** | **ORF5 RFLP** | **ORF5 lineage** | **"-1 PRF" signal**  **codon sequence** | **GenBank**  **accession number** |
| --- | --- | --- | --- | --- | --- | --- |
| ISU15-4143-GA | MARC-145 | IA | 1-4-4 | L1C | CGG | ON053119 |
| ISU15-13621-2 | MARC-145 | NC | 1-7-4 | L1A | TGG | ON053120 |
| ISU15-15871-B | MARC-145 | NE | 1-4-4 | L1C | TGG | ON053121 |
| ISU15-18026 | MARC-145 | IA | 1-7-4 | L1A | TGG | ON053122 |
| ISU15-28864-GA | MARC-145 | IA | 1-18-2 | L1G | TGA | ON053123 |
| ISU15-29455 | MARC-145 | IA | 1-7-4 | L1A | TGG | ON053124 |
| ISU15-37476-A | MARC-145 | SD | 1-26-2 | L1G | TGA | ON053125 |
| ISU15-49649 | MARC-145 | unknown | 1-8-4 | L1H | TGG | ON053126 |
| ISU15-77874-2 | MARC-145 | IN | 1-7-4 | L1A | TGG | ON053127 |
| ISU15-78096-2 | MARC-145 | IA | 1-32-4 | L1A | TGG | ON053128 |
| ISU16-8869 | MARC-145 | unknown | 1-21-4 | L1A | TGA | ON053129 |
| ISU16-31196 | MARC-145 | IA | 1-3-4 | L1C | TGG | ON053130 |
| ISU16-42414 | MARC-145 | IA | 1-7-4 | L1A | TGG | ON053131 |
| ISU16-48574-GA | MARC-145 | OH | 1-26-2 | L1G | TGA | ON053132 |
| ISU16-59538 | MARC-145 | IA | 1-7-4 | L1A | TGG | ON053133 |
| ISU16-62081-3 | MARC-145 | IA | 1-3-3 | L1C | CGG | ON053134 |
| ISU17-5531-GA | MARC-145 | IA | 1-16-4 | L1B | TGG | ON053135 |
| ISU17-32796 | MARC-145 | IA | 1-7-4 | L1A | TGA | ON053136 |
| ISU17-55510 | MARC-145 | NC | 1-7-4 | L1A | TGG | ON053137 |
| ISU17-77143-GE | MARC-145 | IA | 1-7-4 | L1A | CGG | ON053138 |
| ISU17-77158 | MARC-145 | OH | 1-2-4 | L1C | TGG | ON053139 |
| ISU17-79210 | MARC-145 | OH | 1-1-4 | L1C | CGG | ON053140 |
| ISU17-83497 | MARC-145 | UT | 1-4-4 | L1A | TGG | ON053141 |
| ISU18-1842 | MARC-145 | KY | 1-2-4 | L9 | CGG | ON053142 |
| ISU18-3303 | MARC-145 | IA | 1-7-4 | L1A | TGG | ON053143 |
| ISU18-4603 | MARC-145 | PA | 1-4-4 | L1A | TGG | ON053144 |
| ISU18-8114-GA | MARC-145 | MO | 1-67-2 | L1C | CAG | ON053145 |
| ISU18-11533-GA | MARC-145 | IA | 1-8-4 | L1H | TAA | ON053146 |
| ISU18-15326-A | MARC-145 | IN | 1-7-4 | L1A | TGG | ON053147 |
| ISU18-17604-C | MARC-145 | IA | 1-8-4 | L1H | TGG | ON053148 |
| ISU18-19644 | MARC-145 | MO | 1-7-4 | L1A | TGG | ON053149 |
| ISU18-22733-GA | MARC-145 | NC | 1-3-4 | L1A | TGG | ON053150 |
| ISU18-35705-1 | MARC-145 | IA | 1-7-4 | L1A | TGG | ON053151 |
| ISU18-41790 | MARC-145 | NC | 1-7-4 | L1A | TGG | ON053152 |
| ISU18-54157-1 | MARC-145 | NC | 1-7-4 | L1A | TGG | ON053153 |
| ISU18-56050-1 | MARC-145 | MO | 1-18-2 | L1B | TGG | ON053154 |
| ISU18-77867-2 | ZMAC | IL | 1-7-3 | L1A | TGG | ON053155 |
| ISU18-81201 | MARC-145 | IN | 1-10-4 | L1A | TGG | ON053156 |
| ISU18-83705 | MARC-145 | IN | 1-3-4 | L1C | CGG | ON053157 |
| ISU19-7269-1 | ZMAC | MO | 1-7-2 | L1A | TGG | ON053158 |
| ISU19-14156-4 | MARC-145 | WI | 1-8-4 | L1H | TGA | ON053159 |
| ISU19-14353-1 | MARC-145 | IN | 1-7-2 | L1A | TGG | ON053160 |
| ISU19-18603-2 | MARC-145 | OH | 1-8-4 | L1H | CGG | ON053161 |
| ISU19-20528-5 | MARC-145 | MO | 1-7-2 | L1A | TGG | ON053162 |
| ISU19-21123-1 | MARC-145 | MO | 1-4-3 | L1A | CGG | ON053163 |
| ISU19-22196-8 | MARC-145 | OH | 2-5-2 | L5 | TGG | ON053164 |
| ISU19-23254-GA | MARC-145 | IA | 1-7-4 | L1A | TGG | ON053165 |
| ISU19-24207-GA | MARC-145 | IN | 1-8-4 | L1Dbeta | TGA | ON053166 |
| ISU19-34501-1 | MARC-145 | OH | 1-3-4 | L1C | CGG | ON053167 |
| ISU19-45041-GA | MARC-145 | IA | 1-57-4 | L1C | TGG | ON053168 |
| ISU19-46464-GA | MARC-145 | IN | 1-1-4 | L1C | TGG | ON053169 |
| ISU19-51785-GA | MARC-145 | IN | 1-7-4 | L1C | TGG | ON053170 |
| ISU19-52678-1 | MARC-145 | IN | 1-3-2 | L1C | CGG | ON053171 |
| ISU19-53220 | MARC-145 | OH | 1-8-4 | L1H | CTG | ON053172 |
| ISU19-59923-1 | MARC-145 | IN | 1-7-4 | L1A | TGG | ON053173 |
| ISU19-66296-1 | MARC-145 | NC | 1-6-4 | L1A | TGG | ON053174 |
| ISU19-68376-1 | ZMAC | IN | 1-3-4 | L1C | CGG | ON053175 |
| ISU19-76521-GA | MARC-145 | IA | 1-7-4 | L1A | TGG | ON053176 |
| ISU19-89830-1 | ZMAC | IA | 1-3-1 | L1C | CGG | ON053177 |
| ISU19-95000-GA | MARC-145 | IA | 1-8-4 | L1H | CGG | ON053178 |
| ISU19-98200-1 | MARC-145 | IN | 1-12-4 | L1Dbeta | CAG | ON053179 |
| ISU20-6121-1 | ZMAC | OK | 1-8-4 | L1H | TGA | ON053180 |
| ISU20-7040-1 | MARC-145 | IA | 1-8-4 | L1H | TGA | ON053181 |
| ISU20-22120-GA | MARC-145 | IA | 1-118-4 | L1C | TGG | ON053182 |
| ISU20-22617-1 | MARC-145 | OK | 1-4-4 | L1H | TGA | ON053183 |
| ISU20-26614-GA | ZMAC | IL | 1-8-4 | L1H | TGG | ON053184 |
| ISU20-27093-GA | ZMAC | OH | 1-7-4 | L1A | TGG | ON053185 |
| ISU20-28102-2 | MARC-145 | MN | 1-7-4 | L1A | CGG | ON053186 |
| ISU20-29147-GC | MARC-145 | OH | 1-12-4 | L1H | TGG | ON053187 |
| ISU20-32315 | MARC-145 | IN | 1-7-4 | L1A | TGG | ON053188 |
| ISU20-42501-1 | MARC-145 | OK | 1-8-4 | L1H | TGA | ON053189 |
| ISU21-1775-GA | MARC-145 | MN | 1-4-4 | L1Cvariant | CGG | ON053190 |
| ISU21-2839 | MARC-145 | IA | 1-4-4 | L1C | CGG | ON053191 |
| ISU21-6022 | MARC-145 | MN | 1-4-4 | L1Cvariant | CGG | ON053192 |
