# Supplementary material for: Molecular characterization of emerging variants of PRRSV in the United States: new features of the -2/-1 programmed ribosomal frameshifting signal in the nsp2 region
Source: Virology. Author manuscript; Available in PMC 2026 Mar 10. (PMC7618843; doi:10.1016/j.virol.2022.06.004)
Supplement: Table S2 [file EMS212717-supplement-Table_S2.docx]

Table S2.1. Dunnett's multiple comparisons test for CGG mutant versus parental virus or other mutants

|  | **Mean Difference^#^** | **95.00% CI of Difference** | **Adjusted P Value** | **Significance** |
| --- | --- | --- | --- | --- |
| CGG versus CAA | 0.1924 | -0.1491 to 0.5339 | 0.4985 | no |
| CGG versus CAG | 0.2926 | -0.04893 to 0.6341 | 0.1181 | no |
| CGG versus CCG | 0.3462 | 0.004653 to 0.6877 | 0.0459 | * |
| CGG versus CGA | 0.1856 | -0.1559 to 0.5271 | 0.5376 | no |
| CGG versus CUG | 0.3192 | -0.02237 to 0.6607 | 0.0748 | no |
| CGG versus UGC | 0.2804 | -0.06108 to 0.6220 | 0.1442 | no |
| CGG versus UGG | -0.1659 | -0.5074 to 0.1757 | 0.6562 | no |
| CGG versus UUA | 1.185 | 0.8430 to 1.526 | <0.0001 | **** |
| CGG versus UUG | 0.8185 | 0.4769 to 1.160 | <0.0001 | **** |
| CGG versus WT | 0.05225 | -0.2893 to 0.3938 | 0.9993 | no |

# Comparison analysis was based on the titers (genomic copies/ml) of P0 viruses harvested from transfected BHK-21 cells.

Table S2.2. Dunnett's multiple comparisons test for UGG mutant versus parental virus or other mutants

|  | **Mean Difference^#^** | **95.00% CI of Difference** | **Adjusted P Value** | **Significance** |
| --- | --- | --- | --- | --- |
| UGG versus CAA | 0.3582 | 0.01564 to 0.7008 | 0.0375 | * |
| UGG versus CAG | 0.4584 | 0.1158 to 0.8010 | 0.0053 | ** |
| UGG versus CCG | 0.5120 | 0.1694 to 0.8546 | 0.0018 | ** |
| UGG versus CGA | 0.3514 | 0.008851 to 0.6940 | 0.0425 | * |
| UGG versus CGG | 0.1748 | -0.1678 to 0.5174 | 0.6054 | no |
| UGG versus CUG | 0.4850 | 0.1424 to 0.8276 | 0.0031 | ** |
| UGG versus UGC | 0.4463 | 0.1037 to 0.7889 | 0.0068 | ** |
| UGG versus UUA | 1.350 | 1.008 to 1.693 | <0.0001 | **** |
| UGG versus UUG | 0.9843 | 0.6417 to 1.327 | <0.0001 | **** |
| UGG versus WT | 0.2181 | -0.1245 to 0.5607 | 0.3665 | no |

# Comparison analysis was based on the titers (genomic copies/ml) of P0 viruses harvested from transfected BHK-21 cells.
