# Supplementary material for: Molecular characterization of emerging variants of PRRSV in the United States: new features of the -2/-1 programmed ribosomal frameshifting signal in the nsp2 region
Source: Virology. Author manuscript; Available in PMC 2026 Mar 10. (PMC7618843; doi:10.1016/j.virol.2022.06.004)
Supplement: Table S3 [file EMS212717-supplement-Table_S3.docx]

Table S3.1. Dunnett's multiple comparisons test for CGG mutant versus parental virus or other mutants

|  | **Mean Difference^#^** | **95.00% CI of difference** | **Adjusted P Value** | **Significance** |
| --- | --- | --- | --- | --- |
| 12 hpi |  |  |  |  |
| CGG versus CAA | 0.1700 | -0.2181 to 0.5581 | 0.8118 | no |
| CGG versus CAG | 1.000 | 0.6119 to 1.388 | <0.0001 | **** |
| CGG versus CCG | 1.113 | 0.7253 to 1.501 | <0.0001 | **** |
| CGG versus CGA | 0.05667 | -0.3314 to 0.4447 | 0.9994 | no |
| CGG versus CUG | 0.5033 | 0.1153 to 0.8914 | 0.0044 | ** |
| CGG versus UGC | 0.8900 | 0.5019 to 1.278 | <0.0001 | **** |
| CGG versus UGG | -0.05667 | -0.4447 to 0.3314 | 0.9994 | no |
| CGG versus UUA | 1.057 | 0.6686 to 1.445 | <0.0001 | **** |
| CGG versus UUG | 1.057 | 0.6686 to 1.445 | <0.0001 | **** |
| CGG versus WT | -0.1100 | -0.4981 to 0.2781 | 0.9835 | no |
|  |  |  |  |  |
| 24 hpi |  |  |  |  |
| CGG versus CAA | 0.1700 | -0.2181 to 0.5581 | 0.8118 | no |
| CGG versus CAG | 1.057 | 0.6686 to 1.445 | <0.0001 | **** |
| CGG versus CCG | 1.113 | 0.7253 to 1.501 | <0.0001 | **** |
| CGG versus CGA | 0.5567 | 0.1686 to 0.9447 | 0.0012 | ** |
| CGG versus CUG | 1.000 | 0.6119 to 1.388 | <0.0001 | **** |
| CGG versus UGC | 1.113 | 0.7253 to 1.501 | <0.0001 | **** |
| CGG versus UGG | -0.1100 | -0.4981 to 0.2781 | 0.9835 | no |
| CGG versus UUA | 1.447 | 1.059 to 1.835 | <0.0001 | **** |
| CGG versus UUG | 1.337 | 0.9486 to 1.725 | <0.0001 | **** |
| CGG versus WT | -0.1667 | -0.5547 to 0.2214 | 0.8270 | no |
|  |  |  |  |  |
| 36 hpi |  |  |  |  |
| CGG versus CAA | 0.8867 | 0.4986 to 1.275 | <0.0001 | **** |
| CGG versus CAG | 1.443 | 1.055 to 1.831 | <0.0001 | **** |
| CGG versus CCG | 1.500 | 1.112 to 1.888 | <0.0001 | **** |
| CGG versus CGA | 0.6667 | 0.2786 to 1.055 | <0.0001 | **** |
| CGG versus CUG | 1.257 | 0.8686 to 1.645 | <0.0001 | **** |
| CGG versus UGC | 1.553 | 1.165 to 1.941 | <0.0001 | **** |
| CGG versus UGG | -0.05667 | -0.4447 to 0.3314 | 0.9994 | no |
| CGG versus UUA | 2.223 | 1.835 to 2.611 | <0.0001 | **** |
| CGG versus UUG | 2.057 | 1.669 to 2.445 | <0.0001 | **** |
| CGG versus WT | 0.000 | -0.3881 to 0.3881 | >0.9999 | no |
|  |  |  |  |  |
| 48 hpi |  |  |  |  |
| CGG versus CAA | 0.8867 | 0.4986 to 1.275 | <0.0001 | **** |
| CGG versus CAG | 1.277 | 0.8886 to 1.665 | <0.0001 | **** |
| CGG versus CCG | 1.887 | 1.499 to 2.275 | <0.0001 | **** |
| CGG versus CGA | 0.7733 | 0.3853 to 1.161 | <0.0001 | **** |
| CGG versus CUG | 1.167 | 0.7786 to 1.555 | <0.0001 | **** |
| CGG versus UGC | 1.167 | 0.7786 to 1.555 | <0.0001 | **** |
| CGG versus UGG | -0.1133 | -0.5014 to 0.2747 | 0.9795 | no |
| CGG versus UUA | 2.167 | 1.779 to 2.555 | <0.0001 | **** |
| CGG versus UUG | 1.830 | 1.442 to 2.218 | <0.0001 | **** |
| CGG versus WT | -0.07333 | -0.4614 to 0.3147 | 0.9992 | no |
|  |  |  |  |  |
| 60 hpi |  |  |  |  |
| CGG versus CAA | 0.7767 | 0.3886 to 1.165 | <0.0001 | **** |
| CGG versus CAG | 1.000 | 0.6119 to 1.388 | <0.0001 | **** |
| CGG versus CCG | 1.330 | 0.9419 to 1.718 | <0.0001 | **** |
| CGG versus CGA | 0.4967 | 0.1086 to 0.8847 | 0.0052 | ** |
| CGG versus CUG | 0.7767 | 0.3886 to 1.165 | <0.0001 | **** |
| CGG versus UGC | 1.000 | 0.6119 to 1.388 | <0.0001 | **** |
| CGG versus UGG | -0.1100 | -0.4981 to 0.2781 | 0.9835 | no |
| CGG versus UUA | 1.833 | 1.445 to 2.221 | <0.0001 | **** |
| CGG versus UUG | 1.550 | 1.162 to 1.938 | <0.0001 | **** |
| CGG versus WT | -0.1667 | -0.5547 to 0.2214 | 0.8270 | no |

# Comparison analysis was based on the titers (TCID50/ml) of each virus at different time points of growth in MARC-145 cells

Table S3.2. Dunnett's multiple comparisons test of UGG mutant versus parental virus or other mutants

|  | **Mean Difference^#^** | **95.00% CI of Difference** | **Adjusted P Value** | **Significance** |
| --- | --- | --- | --- | --- |
| 12 hpi |  |  |  |  |
| UGG versus CAA | 0.2267 | -0.1614 to 0.6147 | 0.5166 | no |
| UGG versus CAG | 1.057 | 0.6686 to 1.445 | <0.0001 | **** |
| UGG versus CCG | 1.170 | 0.7819 to 1.558 | <0.0001 | **** |
| UGG versus CGA | 0.1133 | -0.2747 to 0.5014 | 0.9795 | no |
| UGG versus CGG | 0.05667 | -0.3314 to 0.4447 | 0.9994 | no |
| UGG versus CUG | 0.5600 | 0.1719 to 0.9481 | 0.0011 | ** |
| UGG versus UGC | 0.9467 | 0.5586 to 1.335 | <0.0001 | **** |
| UGG versus UUA | 1.113 | 0.7253 to 1.501 | <0.0001 | **** |
| UGG versus UUG | 1.113 | 0.7253 to 1.501 | <0.0001 | **** |
| UGG versus WT | -0.05333 | -0.4414 to 0.3347 | 0.9994 | no |
|  |  |  |  |  |
| 24 hpi |  |  |  |  |
| UGG versus CAA | 0.2800 | -0.1081 to 0.6681 | 0.2779 | no |
| UGG versus CAG | 1.167 | 0.7786 to 1.555 | <0.0001 | **** |
| UGG versus CCG | 1.223 | 0.8353 to 1.611 | <0.0001 | **** |
| UGG versus CGA | 0.6667 | 0.2786 to 1.055 | <0.0001 | **** |
| UGG versus CGG | 0.1100 | -0.2781 to 0.4981 | 0.9835 | no |
| UGG versus CUG | 1.110 | 0.7219 to 1.498 | <0.0001 | **** |
| UGG versus UGC | 1.223 | 0.8353 to 1.611 | <0.0001 | **** |
| UGG versus UUA | 1.557 | 1.169 to 1.945 | <0.0001 | **** |
| UGG versus UUG | 1.447 | 1.059 to 1.835 | <0.0001 | **** |
| UGG versus WT | -0.05667 | -0.4447 to 0.3314 | 0.9994 | no |
|  |  |  |  |  |
| 36 hpi |  |  |  |  |
| UGG versus CAA | 0.9433 | 0.5553 to 1.331 | <0.0001 | **** |
| UGG versus CAG | 1.500 | 1.112 to 1.888 | <0.0001 | **** |
| UGG versus CCG | 1.557 | 1.169 to 1.945 | <0.0001 | **** |
| UGG versus CGA | 0.7233 | 0.3353 to 1.111 | <0.0001 | **** |
| UGG versus CGG | 0.05667 | -0.3314 to 0.4447 | 0.9994 | no |
| UGG versus CUG | 1.313 | 0.9253 to 1.701 | <0.0001 | **** |
| UGG versus UGC | 1.610 | 1.222 to 1.998 | <0.0001 | **** |
| UGG versus UUA | 2.280 | 1.892 to 2.668 | <0.0001 | **** |
| UGG versus UUG | 2.113 | 1.725 to 2.501 | <0.0001 | **** |
| UGG versus WT | 0.05667 | -0.3314 to 0.4447 | 0.9994 | no |
|  |  |  |  |  |
| 48 hpi |  |  |  |  |
| UGG versus CAA | 1.000 | 0.6119 to 1.388 | <0.0001 | **** |
| UGG versus CAG | 1.390 | 1.002 to 1.778 | <0.0001 | **** |
| UGG versus CCG | 2.000 | 1.612 to 2.388 | <0.0001 | **** |
| UGG versus CGA | 0.8867 | 0.4986 to 1.275 | <0.0001 | **** |
| UGG versus CGG | 0.1133 | -0.2747 to 0.5014 | 0.9795 | no |
| UGG versus CUG | 1.280 | 0.8919 to 1.668 | <0.0001 | **** |
| UGG versus UGC | 1.280 | 0.8919 to 1.668 | <0.0001 | **** |
| UGG versus UUA | 2.280 | 1.892 to 2.668 | <0.0001 | **** |
| UGG versus UUG | 1.943 | 1.555 to 2.331 | <0.0001 | **** |
| UGG versus WT | 0.04000 | -0.3481 to 0.4281 | 0.9996 | no |
|  |  |  |  |  |
| 60 hpi |  |  |  |  |
| UGG versus CAA | 0.8867 | 0.4986 to 1.275 | <0.0001 | **** |
| UGG versus CAG | 1.110 | 0.7219 to 1.498 | <0.0001 | **** |
| UGG versus CCG | 1.440 | 1.052 to 1.828 | <0.0001 | **** |
| UGG versus CGA | 0.6067 | 0.2186 to 0.9947 | 0.0003 | *** |
| UGG versus CGG | 0.1100 | -0.2781 to 0.4981 | 0.9835 | no |
| UGG versus CUG | 0.8867 | 0.4986 to 1.275 | <0.0001 | **** |
| UGG versus UGC | 1.110 | 0.7219 to 1.498 | <0.0001 | **** |
| UGG versus UUA | 1.943 | 1.555 to 2.331 | <0.0001 | **** |
| UGG versus UUG | 1.660 | 1.272 to 2.048 | <0.0001 | **** |
| UGG versus WT | -0.05667 | -0.4447 to 0.3314 | 0.9994 | no |

# Comparison analysis was based on the titers (TCID50/ml) of each virus at different time points of growth in MARC-145 cells
